# The Instructed Task-Switch Evaluation Effect: Is the Instruction to Switch Tasks Sufficient to Dislike Task Switch Cues?

**DOI:** 10.5334/joc.90

**Published:** 2020-01-07

**Authors:** Pieter Van Dessel, Baptist Liefooghe, Jan De Houwer

**Affiliations:** 1 Ghent University, BE

**Keywords:** Social cognition, Action, Cognitive Control

## Abstract

It is often argued that people dislike situations in which there is conflict requiring cognitive control, possibly because it is effortful to resolve this conflict. In a recent study, Vermeylen, Braem, and Notebaert (2019) provided evidence for this idea in the context of task switching. They observed that participants evaluated cues signaling a task switch more negatively than cues signaling a task repetition in a task switching paradigm. The present study examined whether this evaluative bias can be observed also on the basis of mere instructions. We instructed participants that two non-words would either signal the requirement to switch or to repeat tasks in an upcoming task switching block, which was actually never administered. In Experiment 1, we did not observe more positive implicit or explicit evaluations of the instructed task repetition compared to the task switch cue. In Experiment 2, participants first completed a task switching block in which a first pair of transition cues were used. We then provided task switching instructions that described the signaling function of a second pair of cues, which would be used in an upcoming (but never administered) second task switching block. Participants showed a clear preference for both instructed and experienced task repetition cues on explicit but not on implicit evaluations. Experiment 3 replicated the instructed task-switch evaluation effect on explicit evaluations in the context of prior task experience (but not without prior experience) and extended it to implicit evaluations. We discuss theoretical implications and potential explanations of this task-switch evaluation effect.

A now common view in research on cognitive control, is that the detection and resolution of conflict is tightly interconnected with evaluative responding (e.g., Botvinick, 2007; Kool, Mcguire, Rosen, & Botvinick, 2010; Verguts & Notebaert, 2009; Silvetti, Seurinck, & Verguts, 2011). On the one hand, the neural circuitry underlying cognitive control and affect regulation partially overlaps (e.g., Botvinick et al., 2001; Shackman et al., 2011). On the other hand, several behavioral studies demonstrated that the conflict between competing responses in cognitive-control tasks, such as the Flanker- (Eriksen & Eriksen, 1974) or the Stroop-task (Stroop, 1935), is often negatively evaluated, both on measures of more controlled (i.e., explicit) evaluation and more automatic (i.e., implicit) evaluation (e.g., Brouillet, Ferrier, Grosselin, & Brouillet, 2011; Cannon, Hayes, & Tipper, 2010; Dreisbach & Fischer, 2012, 2015; Fritz & Dreisbach, 2013, 2015; Schouppe, De Houwer, Ridderinkhof, & Notebaert, 2012; Schouppe et al., 2015).

Recently, Vermeylen, Braem, and Notebaert (2019) provided evidence that not only conflict between responses is negatively evaluated, but also conflict between tasks. In two experiments, participants first completed a task switching procedure in which one transition cue (e.g., the word “REPEAT”) probed participants to repeat the same task as on the previous trial and another transition cue (e.g., the word “SWITCH”) probed them to perform a different task. Response conflict is a key feature of task switching in task switching procedures that require participants to rapidly switch between different actions. Moreover, it is well established that such task switching is demanding (see Van Loy, Liefooghe, & Vandierendonck, 2010, for a detailed discussion). Task switches therefore typically induce a performance cost over task repetitions, which is referred to as the switch cost (see Kiesel et al., 2010; Koch et al., 2010; Monsell, 2003; Vandierendonck, Liefooghe, & Verbruggen, 2010, for reviews). Following the task switching block, Vermeylen et al. (2019) probed participants’ implicit evaluation of the cues that had indicated an upcoming task switch compared to the cues that had indicated an upcoming task repetition. In an evaluative priming procedure, transition cues served as primes that were presented prior to Chinese ideographs that participants categorized as positive or negative. Overall, participants provided more positive responses in the context of repetition than in the context of switch cue primes, indicating more negative implicit evaluation of the latter primes. This finding was observed not only when the transition cues were the words “REPEAT” and “SWITCH” but also when non-words were used, which were arbitrarily related to a particular transition on the basis of instructions.

Evaluative effects of response and task conflicts on stimulus evaluations, are reminiscent of a well-established phenomenon in social-psychology research, namely that changes in the evaluation of a stimulus can occur on the basis of the repeated performance of distinct actions that are emitted in response to that stimulus (see Van Dessel, Hughes, & De Houwer, 2019, for a review). For instance, participants who repeatedly approach a certain stimulus and avoid another stimulus typically develop a preference for the approached stimulus over the avoided stimulus (Kawakami, Phills, Steele, & Dovidio, 2007). These effects have often been considered to result from the automatic formation of mental associations that occurs on the basis of the repeated pairing of actions and stimuli (e.g., Phills, Kawakami, Tabi, Nadolny, & Inzlicht, 2011). Likewise, the results of Vermeylen et al. (2019) can be interpreted in terms of the automatic formation of an association between a representation of a word signaling the demand to switch tasks and a representation of the act of switching, which is effortful and therefore negatively evaluated (Vermeylen et al., 2019; see also Kool et al., 2010).

Recent studies, however, have challenged the idea that evaluative effects are underlain by associations formed through repeated practice (see De Houwer, Van Dessel, & Moran, in press, & Corneille & Stahl, 2019, for reviews). An important observation in this debate is that evaluative effects can also be induced on the basis of mere instructions describing contingencies of stimuli and actions, without overtly practicing these contingencies (Van Dessel, De Houwer, Gast, Smith, & De Schryver, 2016; Van Dessel, Gawronski, Smith, & De Houwer, 2017). For instance, Van Dessel, De Houwer, Gast, and Smith (2015) instructed participants to approach one non-word and avoid another non-word. Following these instructions participants showed a preference for the former word both on explicit evaluation measures, such as self-reported liking ratings, and measures of more automatic (i.e., implicit) evaluations such as the Evaluative Priming Task (Fazio, Sanbonmatsu, Powell, & Kardes, 1986) or the Implicit Association Test (IAT; Greenwald, McGhee, & Schwartz, 1998). These and similar findings (Van Dessel, De Houwer, & Gast, 2016; Van Dessel, Hughes, & De Houwer, 2018) are in line with the hypothesis that evaluative effects reflect the formation of propositions or beliefs in memory that are integrated in evaluation via inferential processes (Van Dessel, Hughes, & De Houwer, 2019; see also Van Dessel, Eder, & Hughes, 2019).

The observation that instructions can mimic the effect of overt practice, has also been echoed in research on cognitive control. For instance, response conflicts can be induced on the basis of newly instructed stimulus-response mappings, which have never been overtly applied before (e.g., Liefooghe, Wenke, & De Houwer, 2012; see Brass, Liefooghe, Braem, & De Houwer, 2017; Meiran, Liefooghe, & De Houwer, 2017, for reviews). Brass et al. (2017; see also Liefooghe & Verbruggen, 2019) argued that instructions are assimilated by translating linguistic information into a *task model*. For simple tasks, the construction of the task model only involves compiling a number of verbally instructed S-R mappings in an action-oriented format (Hartstra et al., 2011; Ruge & Wolfensteller, 2010). For more complex tasks (Bhandari & Duncan, 2014; Cole, Laurent, & Stocco, 2013; Dumontheil et al., 2011), the construction of the task model is more complicated. The different sets of relevant rules need to be structured, which involves creating a hierarchical structure and information chunking (Bhandari & Duncan, 2014; Duncan et al., 1996, 2008; Verbruggen, McLaren, Pereg, & Meiran, 2018). Based on this task model, control parameters are implemented, which guide initial task performance (e.g., Chein & Schneider, 2012).

Based on the aforementioned considerations, the question arises whether mere instructions can also induce evaluative effects of cognitive control. To investigate this question, the present study examines whether the relative disliking of task switching cues that was observed in the study by Vermeylen et al. (2019) can be observed based on mere instructions or requires actual experience with the task switching procedure and the transition cues. If instructions are sufficient to produce evaluative effects similar to those reported by Vermeylen and colleagues, then this would suggest that the initial task model created on the basis of instructions, not only includes a representation of the components of a task but also evaluative properties associated with the task or situation described by these instructions.

## Experiment 1

Experiment 1 adapted the design of Van Dessel et al. (2015), which was developed to assess whether instructions to approach or avoid novel stimuli (e.g., non-words) can produce changes in implicit and explicit evaluations of these stimuli. We first provided instructions that two non-words would signal a task switch or a task repetition in an upcoming task switching block. Participants then completed an IAT that probed implicit evaluation of the two non-words and rated their explicit liking of the non-words. Finally, participants were informed that they would not actually complete the task switching block. The central question was whether the non-word that was instructed to signal as a task switch would be evaluated more negatively than the non-word instructed to signal a task repetition (as indexed by responses on implicit and explicit evaluation measures) even when participants never performed any task switching.

### Method

#### Participants

A total of 65 English-speaking volunteers participated online via the Prolific Academic website (https://prolific.ac). The experiment was programmed in Inquisit 4.0 and hosted via Inquisit Web (Millisecond Software, Seattle, WA). In accordance with Vermeylen et al. (2019), sample size was determined using sequential Bayesian hypothesis testing by increasing the sample until a decisive Bayes Factor (BF) larger than 6 (or <1/6) was obtained in the crucial within-subjects *t*-test (Schönbrodt, Wagenmakers, Zehetleitner, & Perugini, 2017). We set the minimal sample size at 60 participants which provides approximately 90% power to find an effect size of *d* = 0.38 (as observed in Experiment 2 of Vermeylen et al., 2019). Note that five additional participants completed the study because they were incorrectly coded as not having performed the experiment by the Prolific Academic software. Sample size increases were planned in steps of 20 participants until a maximum of 120 participants had been tested (providing approximately 90% power to observe a small effect size of *d* = 0.25) but proved unnecessary. This data collection plan was pre-registered on the Open Science Framework website prior to data-collection together with the study design and data-analysis plan. The pre-registered plan and all code and data are available at https://osf.io/ubp6c/.

In line with our pre-registered analysis plan and with the standard treatment of data in online evaluative learning studies (e.g., Van Dessel, De Houwer, Gast, Smith, et al., 2016), we excluded data from participants who (a) did not fully complete all questions and tasks (1 participant; i.e., 1.54%), (b) had IAT error rates for any of the IATs above 30% across the entire task, or above 40% for any one of the four critical blocks (0 participants), or (c) completed more than 10% of IAT trials faster than 400ms (2 participants; i.e., 3.08%). Analyses were performed on the data of 62 participants (40 women, mean age = 27, *SD* = 7).

#### Procedure

After providing informed consent and completing demographic questions, participants learned that the experiment consisted of two parts. The first part would involve a short categorization task and the second part would involve a task switching block in which they needed to perform two tasks at virtually the same time. Participants then received information about the task switching block. Instructions were taken from Vermeylen et al. (2019) and adapted to fit the purposes of the current experiment. First, information was provided about the two constituting tasks:

*For the dual task, a stimulus will be presented on each trial. This stimulus will be a noun referring to an object or an animal (e.g., the word ‘COW’ or the word ‘CHAIR’)*.

*On each stimulus you will have to perform one of two tasks*:

- *decide whether the noun refers to an object or a living animal (e.g., COW => animal, SOAP => object). This is the Alive task*.
  or
- *decide whether the noun refers to something smaller or larger than a basketball (e.g., COW => larger, SOAP => smaller). This is the Size task*.

*You will perform both tasks by using the same response keys ‘Y’ and ‘B’ on the keyboard. Depending on the task at hand, ‘Y’ means animal or smaller than a basketball and ‘B’ means object or larger than a basketball. During the experiment, we will present labels at the top of the screen that help you remember which keys go together with which categories. An example will be provided later on*.

Second, participants were informed about the task switch and task repetition cues:

*The most crucial aspect of this dual task is that you correctly infer which task to perform (Alive OR Size task) on each trial*.

*On the very FIRST trial of the experiment, either the word ALIVE or the word SIZE will be presented next to the target noun (e.g., ALIVE COW). These additional words indicate which task you should perform on that first trial*.

*Importantly, however, on all the following trials the target nouns will be accompanied with either the word YOVIN or the word AFUBU and the following rules apply*:

- *YOVIN means that you need to switch tasks and thus perform the other task compared to the previous trial*.
- *AFUBU means that you need to repeat the same task as on the previous trial*.

*In other words, the relevant task is cued with the word Alive or Size on the very first trial, but on the subsequent trials you have to switch or repeat tasks depending on the word that is presented along with the target noun*.

*It is VERY IMPORTANT that you memorize the aforementioned rules, as only then you will be able to infer which task to perform on each trial*.

After reading the instructions, participants indicated what YOVIN and AFUBU would mean in the task switching block by selecting “it means that I need to switch tasks”, “it means that I need to repeat the previous task”, or “I don’t remember”. If they answered incorrectly, they saw the task switching block instructions again. The non-words YOVIN and AFUBU were also used as stimuli by Vermeylen et al. (2019). It was counterbalanced between participants whether (1) YOVIN or AFUBU was described as the task switch or repetition cue and whether (2) participants first learned about YOVIN or AFUBU.

After correctly answering the instruction check, participants completed an IAT measuring implicit evaluations of the two non-words. Participants categorized eight positive and eight negative words (e.g., wonderful, evil) as ‘positive’ or ‘negative’ and the two non-words YOVIN and AFUBU (presented in different fonts) as their respective names. The IAT followed the procedure described in more detail by Van Dessel, Gawronski, et al. (2016, Experiment 2). In two experimental blocks of 64 trials each, the word YOVIN and positive attributes shared one response key and AFUBU and negative attributes shared a second response key. The IAT block order (i.e., whether it started with the block in which YOVIN shared a key with positive or negative attributes) was counterbalanced between participants.

Next, participants provided ratings of how pleasant or unpleasant they found each of the two non-words. Participants gave their ratings on Likert scales ranging from 1 (extremely unpleasant) to 9 (extremely pleasant).

Finally, participants completed two manipulation check questions that asked what we had instructed them to do when seeing the words YOVIN and AFUBU in the task switching block. Response options were: “YOVIN/AFUBU means that I need to switch tasks”, “YOVIN/AFUBU means that I need to repeat the previous task”, and “I don’t remember”.

### Results

IAT scores were calculated on the basis of participants’ IAT performance using the D4-algorithm (Greenwald, Nosek, & Banaji, 2003) so that positive scores indicate a preference for the task repetition cue over the task switch cue. The split-half reliability of the IAT scores, calculated on the basis of an odd-even split, was *r*(60) = .77. Crucially, a planned one-tailed within-subjects *t*-test indicated that the IAT score was not significantly greater than zero (*M* = –0.07, *SD* = 0.47), *t*(61) = –1.24, *p* = .89, *d_z_* = –0.16, 95% CI diff = [–0.17, Inf] (Table 1). The Bayes factor with Cauchy prior at 0.38 (effect observed in Vermeylen et al., 2019, Experiment 2), indicates that the data provide strong evidence for the absence of this effect, BF_01_ = 8.35. We also performed an ANOVA on IAT scores that included the method factors Identity of the Task Switch Cue (YOVIN or AFUBU), Order of Information about the Task Switch Cue (first or second), and IAT Order (Same key response for positive attributes and switch cue in first or second critical block). We observed a significant effect of Identity of the Task Switch Cue, indicating that participants preferred YOVIN over AFUBU, *F*(1,54) = 11.31, *p* = .001, *η^2^* = 0.14. The intercept was not significant, *F*(1,54) = 2.54, *p* = .12, *η^2^* = 0.03.

**Table 1:** Means and SDs for evaluations of transition cues in Experiments 1–3.

	Explicit Evaluation M (SD)	Implicit Evaluation M (SD)

**Experiment 1**		
Instructed repetition cue	4.84 (1.53)	–0.07 (0.41) (IAT: relative to switch cue)
Instructed switch cue	5.19 (1.63)	
**Experiment 2**		
Instructed repetition cue	5.16 (1.66)	0.57 (0.16)
Instructed switch cue	4.36 (1.46)	0.57 (0.15)
Experienced repetition cue	5.49 (1.86)	0.59 (0.16)
Experienced switch cue	4.64 (1.80)	0.57 (0.17)
**Experiment 3**		
*Prior experience condition*		
Instructed repetition cue	5.23 (1.57)	0.58 (0.17)
Instructed switch cue	4.83 (1.37)	0.53 (0.16)
*No prior experience condition*		
Instructed repetition cue	5.14 (1.37)	0.55 (0.17)
Instructed switch cue	5.07 (1.45)	0.56 (0.16)

Explicit evaluation scores were computed by subtracting the liking rating for the task switch cue from the liking rating for the task repetition cue such that a positive value indicates a relative preference for the task repetition cue. This score was not significantly greater than zero (*M* = –0.35, *SD* = 2.96), *t*(61) = –0.94, *p* = .83, *d_z_* = –0.12, 95% CI diff = [–0.98, Inf], BF_01_ = 7.27. Similar to IAT scores, the 2 × 2 × 2 ANOVA on liking rating scores also revealed a significant effect of Identity of the Task Switch Cue, *F*(1,54) = 9.29, *p* = .004, *η^2^* = 0.13, but no significant intercept, *F*(1,54) = 1.32, *p* = .26, *η^2^* = 0.02.

Analyses for IAT and explicit evaluation scores revealed the same data pattern when excluding the data from 11 participants (i.e., 17.71%) who made at least one error in the manipulation check questions.

### Discussion

Experiment 1 indicates that participants do not exhibit more negative implicit or explicit evaluations of non-words that are instructed to signal a task switch compared to non-words that are instructed to signal a task repetition. Given the results of Vermeylen et al. (2019), this finding might indicate that experiencing a task switching procedure is necessary to dislike task switching cues. Task switching, however, constitutes a rather artificial situation and the additional effort needed on task switches compared to task repetitions may be difficult to derive on the basis of instructions. Moreover, participants might evaluate the task switching demands (i.e., inferring on each trial the task to perform on the basis of a transition cue) as being effortful and negative as a whole, with the difference in effort between task repetitions and task switches, being too futile to induce a difference in evaluation of the transformation cues. Based on these considerations the possibility arises that task switching instructions can produce a preference for task repetition cues only when there is prior experience with the application of older task switching instructions. This was tested in Experiments 2 and 3.

## Experiment 2

In Experiment 2, we included a task switching block that followed the procedures outlined by Vermeylen et al. (2019). In contrast to their study, however, we used two non-word pairs and provided instructions about one pair after the task switching block. Specifically, we first presented participants with a task switching block in which a first pair of non-words served as transition cues. Next, it was instructed that a second pair of non-words would serve as transition cues in an upcoming task switching block. Following these instructions, participants completed an Affective Misattribution Procedure (AMP; Payne, Cheng, Govorun, & Stewart, 2005). In contrast to the IAT, the AMP employs an evaluative priming procedure and can be used to measure implicit evaluations of more than two target stimulus categories simultaneously (as was currently the case: experienced switch cue, experienced repetition cue, instructed switch cue and instructed repetition cue). Finally, participants rated the explicit liking of all four non-words. The central question was whether participants would exhibit more negative implicit or explicit evaluations for the non-words that were experienced or instructed to signal task switches compared to the non-words signaling task repetitions.

### Method

#### Participants

A total of 202 English-speaking volunteers participated online via the Prolific Academic website. In line with Experiment 1, we used a pre-registered sequential Bayesian hypothesis testing procedure but now set our maximum sample size at 200 participants to ensure that results would be informative also about the presence or absence of small effect sizes (we had 90% power to find an effect size of *d* = 0.20). We excluded data from participants who (1) did not fully complete all questions and tasks (7 participants; i.e., 3.47%) and (2) responded with the same key for more than 90% of trials in the AMP (8 participants; i.e., 5.47%) (in accordance with Vermeylen et al., 2019). Analyses were performed on the data of 187 participants (93 women, mean age = 29, *SD* = 8).

#### Procedure

After providing informed consent and completing demographic questions, participants were informed that the experiment consisted of three parts: (1) a task switching block in which they needed to perform two tasks at virtually the same time, (2) a task in which they needed to evaluate Chinese ideographs, and (3) the same task switching block as before. Similar to Experiment 1, participants then received information regarding the task switching block and the pair of task switch cues in the first task switching block. Specifically, participants were informed about the cueing function that the words BAYIR and YIRPS would have in the task switching block. They were then asked to report this function in the instruction check. Given the relative preference for one of the non-words in Experiment 1, we used other non-words to function as cues: BAYIR, YIRPS, WOZED, and NUILE. These non-words were selected on the basis of a pilot study in which 50 Prolific Academic participants rated these non-words as the most neutral of 50 five-letter non-words on a 7-point rating scale with 4 as neutral mid-point and also rated them as highly unfamiliar. It was counterbalanced between participants whether BAYIR or YIRPS was described as the task switch or repetition cue and whether participants first received information about BAYIR or YIRPS.

After completing the instruction check, participants completed 10 practice trials in which they categorized words by pushing the Y keyboard key assigned to the category “larger than a basketball” or the B keyboard key assigned to the category “smaller than a basketball” (size task). Next, participants used the same keys to complete 10 practice trials in which they categorized words as living or nonliving (alive task). Participants then completed 20 practice trials in which they switched between the tasks as indicated by the task repetition and task switch cues. Finally, participants completed 160 task switching block trials. All stimuli and task switching block parameters were identical to Vermeylen et al. (2019).

Next, participants received instructions that another task switching block would follow in which two other words would indicate whether they needed to repeat or switch tasks. They were informed that WOZED meant that they would need to switch tasks and thus perform the other task compared to the previous trial and NUILE meant that they would need to repeat the same task as on the previous trial (or vice versa – counterbalanced assignment of words to cueing function). Participants then completed the instruction check questions about the cueing function of the two non-words.

Participants were then informed that, before doing the task switching block, they would need to perform another task. This task was a standard AMP constituting two blocks of 80 trials in which participants categorized Chinese ideographs as Unpleasant (D key) or Pleasant (K key). Crucially, however, on each trial, the ideographs were presented back-ward masked and only for a short duration of time (100 ms) and were preceded by one of the four cue words (YIRPS, BAYIR, WOZED, or NUILE), presented for 75 ms. In line with standard recommendations, participants were informed that the words that flashed before the Chinese ideographs can sometimes influence people’s responses and that they should try their best not to let the words influence their judgments of the Chinese ideographs.

Next, participants rated how pleasant or unpleasant they considered each of the four non-words (randomized order) and completed four manipulation check questions that probed the (instructed or experienced) cueing function of the non-words in the task switching blocks. Participants also rated (1) how difficult the task switching block was, (2) how unpleasant this difficult task was, (3), how difficult the task switches were, and (4) how unpleasant these task switches were. Responses were given on 9-point Likert scales.

Finally, participants were informed that they did not need to perform a second task switching block and they were thanked for participation.

### Results

A first AMP score was calculated by subtracting the number of pleasant responses on AMP trials with the experienced task switch cue as prime from the number of pleasant responses on AMP trials with the experienced task repetition cue as prime. The split-half reliability of this AMP score was *r*(179) = .47. In contrast to results of Vermeylen et al. (2019), the planned one-tailed within-subjects *t*-test indicated that the AMP score was not significantly greater than zero (*M* = 0.02, *SD* = 0.16), *t*(180) = 1.55, *p* = .061, *d_z_* = 0.11, 95% CI diff = [–0.01, Inf], BF_01_ = 1.16. A second AMP score was calculated by subtracting the number of pleasant responses on AMP trials with the instructed task switch cue as prime from the number of pleasant responses on AMP trials with the instructed task repetition cue as prime. The split-half reliability of this AMP score was *r*(179) = .47. The planned one-tailed within-subjects *t*-test indicated that this AMP score was also not significantly greater than zero (*M* = 0.00, *SD* = 0.17), *t*(180) = 0.13, *p* = .45, *d_z_* = 0.01, 95% CI diff = [–0.02, Inf], BF_01_ = 5.98. ANOVAs on both AMP scores that included the method factors Identity of the Task Switch Cue and Order of Information about the Task Switch Cue did not reveal any significant effects, *F*s < 2.68, *p*s > .10, *η^2^*s < 0.02.

Explicit evaluation scores were computed by subtracting the liking rating for the experienced and instructed task switch cue from the liking rating for the corresponding task repetition cue. In contrast to AMP scores, explicit evaluation scores for the experienced cues revealed a preference for the repetition cue (*M* = 0.84, *SD* = 2.82), *t*(180) = 4.01, *p* < .001, *d_z_* = 0.30, 95% CI diff = [0.49, Inf], BF_10_ = 446.85. Importantly, this preference was also observed on liking rating scores for the instructed cues (*M* = 0.80, *SD* = 2.45), *t*(180) = 4.40, *p* < .001, *d_z_* = 0.33, 95% CI diff = [0.50, Inf], BF_10_ = 1868.69. ANOVAs on liking rating scores revealed significant intercepts for instructed and experienced cues, *F*s > 15.70, *p*s < .001, *η^2^*s > 0.08, and revealed an effect of Identity of the Task Switch Cue for instructed cues, *F*(1,173) = 5.17, *p* = .024, *η^2^* = 0.03, indicating a preference for WOZED over NUILE. Analyses for IAT and liking rating scores revealed the same data pattern when excluding the data from 39 (i.e., 31.49%) or 57 (i.e., 21.55%) participants who made at least one error in the manipulation check questions for instructed or experienced cues, respectively. That is, we observed no effect on AMP scores for experienced, *t*(108) = 1.46, *p* = .073, or instructed cues, *t*(108) = 1.58, *p* = .058, but we did observe an effect on explicit rating scores for experienced and instructed cues, *p*s < .001.

Performance in the task switching block revealed typical switch costs such that participants made more errors on switch than on repetition trials (*M_Diff_* = 0.04, *SD* = 0.07) *t*(180) = 7.44, *p* < .001, and participants performed better on repetition than on switch trials as indexed by the difference in mean latencies for task switch compared to task repetition trials divided by participants’ mean latency overall (*M_Diff_* = 0.07, *SD* = 0.12) *t*(180) = 7.67, *p* < .001. In line with Vermeylen et al. (2019), we also performed correlational analyses that examined the relation between participants’ task switching effect and implicit and explicit evaluations (of instructed and experienced cues). We observed a significant correlation between the explicit rating score for experienced cues with switch costs on latencies, *r(*179) = 0.25, *p* < .001, indicating a bigger explicit preference for task repetition cues for participants with higher switch costs. This correlation was not observed for the instructed cues, *r(*179) = 0.11, *p* = .14. We observed no other significant correlations between explicit or implicit scores with switch costs on latencies or errors, *r*s < 0.13, *p*s > .095. For exploratory purposes, we also correlated AMP and explicit rating scores with participants’ rated difficulty of (switching in) the task switching block and its pleasantness. We did not observe any significant correlations, *r*s < 0.13, *p*s > .080.

### Discussion

The explicit ratings in Experiment 2 indicate a clear preference for task repetition over task switch cues. In contrast, the AMP scores did not indicate such difference (cfr. Vermeylen et al., 2019). When considering the results of Experiments 1 and 2, the conclusion could be drawn that a task-switch evaluation effect can be induced on the basis of instructions, only when some prior experience with task switching is provided. In Experiment 3, we aim to strengthen this conclusion by directly manipulating the opportunity to experience of task switching.

## Experiment 3

In Experiment 3, one group of participants completed the same procedure as in Experiment 2 (i.e., instructions for experienced cues, task switching block with experienced cues, instructions for instructed cues, evaluation). A second group completed the same procedure as in Experiment 1 (i.e., instructions for instructed cues, evaluation; they did not receive experience with the task switching block prior to evaluation). This experiment thus consists of a replication of Experiment 1, which found no instructed task-switch evaluation effect in the absence of prior task switching experience, and Experiment 2, which did find an effect when participants had prior experience with the task-switching block. Importantly, this set-up allows us to more directly examine whether the instructed task-switch evaluation effect depends on prior task switching experience.

We also made two additional changes compared to Experiment 2. First, after the evaluation phase, all participants completed a task switching block with instructed cues. This allow us to probe differential performance in this block for participants who did and who did not receive prior task switching experience (e.g., due to differences in understanding or implementation of the instructions). Second, participants only provided evaluations of the two instructed cues (and not of the experienced cues) in the evaluation phase of the experiment. This has the advantage that (1) evaluation of experienced cues does not bias evaluation of the instructed cues and (2) fewer targets are used in the AMP which could improve the reliability of the AMP evaluation scores.

### Method

#### Participants

A total of 280 English-speaking volunteers participated online via the Prolific Academic website. We again used a pre-registered sequential Bayesian hypothesis testing procedure, but this time the stopping rule depended on the results of two separate *t*-tests probing the presence or absence of an instructed task-switch evaluation effect on explicit evaluations in the condition with prior task experience and in the condition without prior task experience. We set our maximum sample size at 280 participants to ensure that results would be informative about the presence or absence of small effect sizes (we had 90% power to find an effect size of *d* = 0.25 in each condition). We excluded data from participants who (1) did not fully complete all questions and tasks (2 participants; i.e., 0.71%) and (2) responded with the same key for more than 90% of trials in the AMP (32 participants; i.e., 11.43%). Analyses were performed on the data of 246 participants (161 women, mean age = 28, *SD* = 7). Participants were randomly assigned to the condition in which they either received experience with the task switching block before (prior experience condition) or after the evaluation phase (no prior experience condition).

#### Procedure

Experiment 3 was identical to Experiment 2 except for the following points. First, only half of the participants first received task switching instructions for experienced cues, then completed the task switching block with these cues, then received task switching instructions for instructed cues, and then completed evaluation tasks. The other participants first received task switching instructions for instructed cues and then completed evaluation tasks. In contrast to Experiment 2, all participants also completed the task switching block with the instructed cues at the end of the experiment.

Second, BAYIR and YIRPS were used as instructed cues (because these words had been rated as highly similar in valence in Experiment 2), whereas WOZED and THUCE were used as experienced cues (in the prior task experience condition). Note that we used the word THUCE instead of NUILE because in Experiment 2, NUILE was rated as more negative than the other non-words. Note that the evaluation tasks involved evaluation of the instructed cues only (i.e., BAYIR and YIRPS).

Third, we did not include exploratory questions at the end of the experiments because responses on these questions did not relate to any of the effects of interest in Experiment 2.

### Results

In line with Experiment 2, for participants in the prior experience condition, explicit evaluation scores revealed a preference for the instructed repetition over the instructed switch cue (*M* = 0.40, *SD* = 2.09), *t*(123) = 2.14, *p* = .017, *d_z_* = 0.19, 95% CI diff = [0.09, Inf], BF_10_ = 3.05. This instructed task-switch evaluation effect was not observed for participants in the no prior experience condition (*M* = 0.08, *SD* = 1.40), *t*(119) = 0.59, *p* = .28, *d_z_* = 0.05, 95% CI diff = [–0.14, Inf], BF_01_ = 2.93. The ANOVA on liking rating scores revealed a significant intercept, *F*(1,236) = 4.23, *p* = .041, *η^2^* = 0.02, but no effect of Prior Experience Condition, *F*(1,236) = 1.79, *p* = .18, *η^2^* = 0.01, or of any of the method factors, *F*s < 0.34, *p*s >.54, *η^2^*s < 0.01.

The split-half reliability of the AMP score was *r*(242) = .67. In contrast to Experiment 2, the AMP score of participants in the prior experience condition was significantly greater than zero, indicating an implicit preference for the instructed repetition cue (*M* = 0.05, *SD* = 0.26), *t*(123) = 2.20, *p* = .015, *d_z_* = 0.20, 95% CI diff = [0.01, Inf], BF_10_ = 3.40. This preference was not observed for participants in the no prior experience condition (*M* = 0.00, *SD* = 0.22), *t*(119) = –0.14, *p* = .55, *d_z_* = 0.01, 95% CI diff = [–0.04, Inf], BF_01_ = 5.34. The ANOVA on AMP scores did not reveal a significant intercept, *F*(1,236) = 2.23, *p* = .13, *η^2^* = 0.01, or an effect of Prior Experience Condition, *F*(1,236) = 2.66, *p* = .10, *η^2^* = 0.01, or of any of the method factors, *F*s < 0.34, *p*s > .54, *η^2^*s < 0.01.

We observed more errors on switch than on repetition trials in the task switching block with instructed cues (performed after evaluation) and in the task switching block with experienced cues (performed before evaluation; only performed by participants in the prior experience condition), *t*s > 6.52, *p*s < .001. In both tasks, we also observed better performance on repetition than on switch trials as indexed by the difference in mean latencies for task switch compared to task repetition trials divided by participants’ mean latency overall, *t*s > 7.17, *p*s < .001. Correlational analyses revealed a significant correlation between the instructed task-switch evaluation effect on AMP scores and the task switch cost observed on error rates for participants with prior experience, *r*(122) = 2.19, *p* = .030. However, no other significant correlations were observed for participants in either condition between the task-switch evaluation effect on AMP or explicit rating scores with performance in the task switching block with instructed cues, *rs* < 0.14, *p*s > .10, or with experienced cues, *rs* < 0.15, *p*s > .10.

To examine possible differences between participants who did and who did not receive task switching experience in remembering or implementing instructions, we also inspected performance in the task switching block with instructed cues as indexed by participants’ overall errors and switch costs. T-tests revealed no significant differences between performance in this task switching block for participants in the no prior experience condition compared to performance in the task switching block with instructed or experienced cues for participants in the prior experience condition, *t*s < 1.36, *p*s > .17.

### Discussion

Experiment 3 replicated the finding that participants who have previously performed a task switching session indicate more positive explicit liking for instructed task repetition over instructed task switch cues. In contrast to Experiment 2, this preference was also observed on AMP scores, which could relate to the fact that AMP scores were more reliable in Experiment 3. In line with the findings of Experiment 1, the instructed task-switch evaluation effect was not observed when participants did not experience task switching before evaluation. Note that the ANOVA results did not confirm that prior task experience condition was a significant moderator of the instructed task-switch evaluation effect. This could, however, be due to the fact that the instruction effect was small, and we had insufficient statistical power to observe a moderation of such a small effect. To examine this issue with more statistical power, we performed ANOVAs that included the data of Experiments 1–3, revealing the expected effect of Prior Experience Condition for the instructed task-switch evaluation effect on explicit, *F*(1,485) = 10.19, *p* = .002, *η^2^* = 0.02, but not implicit evaluations, *F*(1,485) = 2.68, *p* = .10, *η^2^* = 0.01.

## General Discussion

Inspired by the recent findings of Vermeylen et al. (2019), we conducted three experiments in which we tested whether merely providing task switching instructions can produce a preference for task repetition over task switch cues. Experiment 1 indicates that mere instructions that ascribe one non-word to a task repetition and one non-word to a task switch function is not sufficient to induce this evaluative bias. In Experiment 2, participants first completed a task switching block that used a first pair of transition cues and then a second pair of transition cues was instructed for an upcoming task switching block. The expected preference for task repetition cues was observed both for experienced and instructed cues on explicit liking ratings but not on the AMP. Experiment 3 replicated the instructed task-switch evaluation effect in the context of prior task experience and the absence of this effect in the context of no prior experience on explicit ratings. Moreover, the instructed task-switch evaluation effect in the context of prior task experience was extended to AMP scores. Taken together, our results suggest that instructions to switch tasks can induce a negative evaluative bias towards task switching cues, albeit only when participants previously experienced task switching. Most probably, a key aspect of this task switching experience is that task switches are experienced as more effortful than task repetitions and this experience is necessary to learn that task switching cues are more negative than task repetition cues (see also Kool et al. 2010; Vermeylen et al., 2019).

It is noteworthy that Experiment 2 found similar effects for instructed and experienced cues (in absolute terms, the effect size was even slightly bigger for instructed cues). This relative power of instructions to induce changes in liking has been observed in previous studies as well (e.g., Kurdi & Banaji, 2017). In research on (operant) evaluative conditioning, which refers to the change in liking that results from the pairing of a stimulus with another valenced stimulus (or action), these findings have fuelled important debates about the mental processes underlying these effects. Most prominently, they questioned the idea that conditioning effects result from the automatic formation of mental associations that occur on the basis of the repeated pairings and supported explanations that draw on the formation of propositions (or beliefs) in memory that are integrated in evaluation via inferential processes (Van Dessel, Hughes, & De Houwer, 2019). Similar propositional processes might also underlie the task-switch evaluation effect. Participants might form the proposition that specific cues will function as task switch or task repetition cues and infer that task repetition cues are more positive. This inference, however, may depend on the expectancy that switching is effortful and thus negative and this might require actual experience of (the difficult nature of) task switching. In the absence of such experience, the average proposition created on the basis of instructions might not be geared towards a disliking of task switching. In fact, the naïve expectancy of the valence of task switching could vary strongly such that some people might even infer that task switching is positive (e.g., because it could prevent boredom). Experience (with the task designed by Vermeylen et al., 2019) might provide new information that reduces this variance by showing that task switching is effortful (e.g., Kool et al., 2010). Note that, from this perspective, it should be possible to (1) design a task switching block in which task switching is not effortful (but e.g., prevents boredom) such that participants might come to prefer experienced task switching cues over task repetition cues and (2) design instructions in which the effortful nature of task switching is emphasized such that an instruction effect can be observed in the absence of any task switching experience.

In line with the previous considerations, it can be argued that when a task model is constructed on the basis of instructions (e.g., Brass et al., 2017), the evaluative connotations of this model are only included when some knowledge is available about which components of the instructed task are effortful. Such prior knowledge can be based on prior experience. However, instructions can also convey such information, thus leading to evaluative effects in the absence of prior experience.

One limitation of our study is that we did not observe strong evidence for evaluative biases on implicit evaluations. The results of the IAT and the AMP indicated substantial evidence (estimated by Bayes Factors) for the absence of a difference in implicit evaluations of task switch cues and task repetition cues in Experiments 1–2. This seems at odds with the results of Vermeylen et al. (2019), who observed that task switch cues were evaluated more negatively when using the transition cues as primes in an evaluative priming procedure. Note, however, that the present study used two measures that are often used to examine evaluations that have certain features of automaticity (e.g., unintentionality: Mann, Cone, Heggeseth, & Ferguson, 2019; see Gawronski & De Houwer, 2014, for a review). The evaluative priming procedure used by Vermeylen et al. (2019) may have differed in this respect. For instance, in contrast to a typical AMP, their task required participants to actively process the prime words by using catch trials and primes were presented for a relative long period of time, ranging from 200ms to 800ms. Although this procedure might have merits for measuring evaluations (see also Fritz & Dreisbach, 2015), these evaluations might be considered less automatic and it is possible that the task-switch evaluation effect can only be observed on this type of evaluations (e.g., because the inferences underlying the effect are not sufficiently automatic: Van Dessel et al., 2019). Another possibility is that (1) prior task experience is necessary for the effect (preventing an IAT effect in Experiment 1) and (2) the procedure used by Vermeylen et al. probed evaluations in a more reliable manner than the AMP (preventing an AMP effect in Experiment 2). Accordingly, Experiment 3 found an instruction effect on AMP scores (in the prior experience condition). This dissociation with Experiment 2 could be related to the higher reliability of AMP evaluation scores observed in Experiment 3 (possibly due to the fact that fewer targets were used in the AMP). Because the Bayes Factor for the AMP effect in Experiment 3 was not very strong (BF_10_ = 3.40), future research should investigate whether this effect is robust and replicable and whether other implicit evaluation measures such as the IAT can observe this change in liking.

## Data Accessibility Statement

All the materials related to this paper (the stimulus list, the data collected, the analysis scripts, and the tools that we used during the analysis) are publicly available at the Open Science Framework (https://osf.io/ubp6c/).
